# Prevalence, Incidence, and Factors Associated with Posttraumatic Stress at Three-Month Follow-Up among New York City Healthcare Workers after the First Wave of the COVID-19 Pandemic

**DOI:** 10.3390/ijerph19010262

**Published:** 2021-12-27

**Authors:** Ari Shechter, Codruta Chiuzan, Yimeng Shang, Gavin Ko, Franchesca Diaz, Hadiah K. Venner, Kaitlin Shaw, Diane E. Cannone, Cara L. McMurry, Alexandra M. Sullivan, Reynaldo R. Rivera, Courtney Vose, Peter A. Shapiro, Marwah Abdalla

**Affiliations:** 1 Department of Medicine, Columbia University Irving Medical Center, New York, NY 10032, USA; as4874@cumc.columbia.edu (A.S.); fd2420@cumc.columbia.edu (F.D.); hkv2001@cumc.columbia.edu (H.K.V.); kes2196@cumc.columbia.edu (K.S.); dew2106@cumc.columbia.edu (D.E.C.); mlc2238@cumc.columbia.edu (C.L.M.); as5068@cumc.columbia.edu (A.M.S.); 2 Institute of Health System Science, Feinstein Institute for Medical Research, Northwell Health, New York, NY 10022, USA; cc3780@cumc.columbia.edu; 3 Department of Biostatistics, Mailman School of Public Health, Columbia University, New York, NY 10032, USA; ys3298@cumc.columbia.edu (Y.S.); wk2343@cumc.columbia.edu (G.K.); 4 New York-Presbyterian Hospital, New York, NY 10032, USA; rrr9001@nyp.org (R.R.R.); cov9012@nyp.org (C.V.); 5 School of Nursing, Columbia University, New York, NY 10032, USA; 6 Department of Psychiatry, Columbia University Irving Medical Center, New York, NY 10032, USA; pas3@cumc.columbia.edu

**Keywords:** healthcare worker, acute stress, posttraumatic stress, COVID-19, mental health

## Abstract

Background: Prevalence, incidence, and factors associated with posttraumatic stress disorder (PTSD) symptoms at follow-up among healthcare workers after the first wave of the COVID-19 pandemic are unknown. Methods: A web survey invitation was sent to healthcare worker listservs at a NYC medical center (April, 2020). The Primary Care (PC)-PTSD questionnaire was used to screen for PTSD symptoms at baseline and then every 2 weeks for 10 weeks. Incidence and prevalence of PTSD symptoms were determined at each time point. Multivariable generalized estimating equation models were performed to investigate the factors associated with a positive PC-PTSD screen at follow-up. Results: Median age (interquartile range) of N = 230 participants was 36 (31–48) years; 79.6% were women; 82.6% worked in COVID-19-focused settings. The prevalence of PTSD symptoms decreased from 55.2% at baseline to 25.0% at 10 weeks (*p* < 0.001). Among participants who had a baseline negative screen for PTSD symptoms, the incidence of PTSD at 10 weeks was 12.2% (p-trend 0.034). In multivariable-adjusted analyses, being a nurse (odds ratio [OR]: 1.70, 95% confidence interval [CI]: 1.06–2.71), female (OR: 3.00, 95% CI: 1.59, 5.72), and working in a COVID-19-focused location (OR: 1.51, 95% CI: 1.02, 2.21) were associated with increased odds of PTSD symptoms at 10-weeks. Conclusions: PTSD symptoms improved over 3 months following the first wave of the COVID-19 pandemic. However, one out of four NYC healthcare workers still had an increased risk for PTSD at 10-weeks. Screening healthcare workers for PTSD symptoms should be considered during the COVID-19 pandemic.

## 1. Introduction

During the COVID-19 pandemic, healthcare workers have been exposed to stressful occupational conditions, including long hours, increased risk of COVID-19 infection, and excessive patient workload. Acute psychological distress among healthcare workers during the pandemic has been described. Cross-sectional studies have demonstrated that the prevalence of acute stress may be as high as 73% [1]. Calls to address and improve healthcare workers’ mental health during the COVID-19 pandemic have increased nationally and internationally including from the World Health Organization [2,3].

The acute experience of natural disasters and public health crises, including infectious disease outbreaks (i.e., severe acute respiratory syndrome [SARS]) [4,5], can induce trauma and symptoms of posttraumatic stress disorder (PTSD) [6], which may arise as early as four weeks after an acute stressor. PTSD has been associated with an increased risk of depression, suicide, chronic diseases, and mortality [6,7,8,9,10]. Data from the World Trade Center Health Registry (*n* = 63,666) demonstrated that first responders exposed to the World Trade Center (WTC) attacks in New York City (NYC) on 11 September 2001 had an increased risk for all-cause and cardiovascular mortality over a 13-year follow-up period [8]. Additionally, data from a longitudinal epidemiological study of 1200 young adults during a 10-year follow-up period demonstrated that individuals with prior trauma and a previous diagnosis of PTSD have an increased risk of developing PTSD in response to subsequent trauma [11]. This highlights that the exacerbation of symptoms of PTSD may be particularly increased in individuals with a previous diagnosis of PTSD.

These findings have particular relevance in the wake of the COVID-19 pandemic, as rates of acute stress among healthcare workers have been high. However, few data [12,13] exist examining the prevalence of and factors associated with PTSD symptoms at follow-up in healthcare workers during the COVID-19 pandemic. One recent multisite study of Emergency Department physicians working in different hospital systems across the US demonstrated that acute stress symptoms decreased from 32.8% to 25.9% when assessed only once 4–6 weeks later [12]. Besides a one-time assessment, that study was also limited by heterogeneity in participants’ levels of acute stress, as some sites had not yet been affected by the pandemic. Therefore, we examined the prevalence and incidence of posttraumatic stress symptoms across a 10-week follow-up period among NYC healthcare workers (physicians, nurses, advanced practice providers, house staff/fellows) working at a large academic center during the initial wave of the COVID-19 pandemic in the spring of 2020, when NYC was the center of the US COVID-19 pandemic [14]. We also examined the factors associated with posttraumatic stress symptoms among participants at follow-up. 

## 2. Materials and Methods

### 2.1. Study Design

Participants were healthcare workers (physicians, nurses, advanced practice providers, house staff/fellows) from a large NYC medical center during the first wave of the COVID-19 pandemic in NYC, described previously [15]. Participants were recruited using a standardized recruitment email that was sent to physicians, advanced practice providers, nurses, and house staff/fellow listservs. The first email for the baseline cross-sectional study was sent on 9 April 2020 (an initial peak of the COVID-19 pandemic in NYC [16,17]), and the first participant enrolled on that date. A total of 1247 participants enrolled in the baseline cross-sectional study, of whom 827 completed all questions. Upon completing the baseline study, participants were given the opportunity to participate in a separate, longitudinal study. A subset of the original participants (*n* = 230) also enrolled separately in the longitudinal follow-up study. Participants who enrolled in the longitudinal follow-up study and were included in this analysis completed additional questionnaires every two weeks for up to three months. During the study period, NYC and New York State had a stay-at-home order in effect [18]. Only healthcare workers and other essential workers were allowed to continue to work. Quarantine was mandatory for anyone exposed to, or infected with, COVID-19. Further, there were structural changes to the hospital workplace environment including conversion of patient wards into makeshift intensive care units at our medical center in order to deal with the influx of patients during the COVID-19 pandemic [19]. The Columbia University Irving Medical Center Institutional Review Board approved the study protocol. All participants provided informed consent. 

### 2.2. Study Survey

The survey had questions about demographics (age, sex, race/ethnicity), clinical roles (physician, nurse, advanced practice providers, house staff/fellows), assignments, assessment of distress due to COVID-19 specific stressors, psychological screens, coping behaviors, and wellness resources desired by healthcare workers [15]. 

### 2.3. Psychological Screen for Acute Stress and Posttraumatic Stress Symptoms

We used the 4-item Primary Care PTSD screen [20] (PC-PTSD, range 0–4; score ≥ 3 indicates a positive screen) as a marker for elevated acute stress symptoms for the baseline assessment, as less than a month had passed since the start of the pandemic and first participant enrollment. The PC-PTSD screen was also repeated at every follow-up assessment (i.e., every two weeks for a total of 10 weeks) to assess the presence of posttraumatic stress symptoms since the prior survey assessment. Positive screens on the PC-PTSD after the 4-week follow-up (i.e., week six and later) were indicative of the presence of posttraumatic stress symptoms and increased risk of PTSD. 

### 2.4. Statistical Analysis

Descriptive statistics were summarized as frequencies (percentages) for the categorical variables and median (interquartile range IQR) for age and number of hours worked in the past week. Based on the clinical relevance and frequency distributions, in the analysis the main outcome of PC-PTSD was categorized into two groups: “positive screen” (≥3) vs “negative screen” (<3). The participant’s race/ethnicity was also dichotomized into “White, non-Hispanic/Latino” vs. “other”. 

We determined the prevalence and incidence of a positive screen on the PC-PTSD at each timepoint. To investigate the factors associated with a positive screen, a generalized estimating equation (GEE) model was applied to accommodate the correlated data of repeated measurements (at baseline and at weeks 2, 4, 6, 8, and 10 of follow-up) with a logit link function and an exchangeable correlation structure. We conducted both univariable and multivariable GEE analyses using preselected variables that may be associated with a positive PC-PTSD screen based on findings from the literature [7,13,14,15]. Baseline covariates included age, sex (female vs. male), race/ethnicity (White, non-Hispanic/Latino vs. other), clinical role (registered nurse [RN] vs. other), clinical setting during the local peak of COVID-19 (working in a COVID-19-focused area, defined as the emergency department, intensive care unit, inpatient or outpatient COVID-19 areas vs. working in a non-COVID-19 area), and number of hours worked over the past week with categories ranging from (1) 0–10 h to (13) more than 120 h. The goodness-of-fit of the multivariable model was assessed using an extension of the Hosmer and Lemeshow statistic for repeated binary observations using predicted deciles of risk. All statistical analyses were performed in R (v. 4.1.0, MathSoft, Seattle, WA, USA) using a two-sided type I error of 0.05.

## 3. Results

A total of 230 healthcare workers who completed the baseline study and agreed to enroll in the longitudinal study were included in the current analysis. Table 1 provides descriptive characteristics for participants enrolled in the longitudinal study at their baseline assessment. 

The median age (IQR) of participants in the longitudinal study was 36 (31–48) years. Participants were 79.6% female, 64.3% White, and 13.2% Hispanic, while 50.0% were nurses and 82.6% worked in COVID-19-focused settings. There were no differences in participant characteristics between those enrolled in the longitudinal study compared to those who only completed the baseline assessment but did not enroll in the longitudinal study (Supplementary Table S1). 

At the baseline assessment, the median (IQR) PC-PTSD score was 3 (1–4) for participants enrolled in the longitudinal study compared to 3 (2–4) for participants not enrolled in the longitudinal study, *p* = 0.116 (Supplementary Table S1). There was no statistically significant difference in the proportion of participants with a positive PC-PTSD screen (PC-PTSD score ≥ 3). Among participants enrolled in the longitudinal study, the proportion with a positive PC-PTSD screen (PC-PTSD ≥ 3) at the baseline assessment was 55.2%. Among participants not enrolled in the longitudinal study, the proportion with a positive PC-PTSD screen at the baseline assessment was 59.1% (*p* = 0.307). We also compared baseline participant characteristics among those who completed the 10-week follow-up assessment (*n* = 88) versus those who did not complete the 10-week follow-up assessment (*n* = 142) (Supplementary Table S2). There were differences in age (younger in those who completed the 10-week follow-up vs. those who did not complete the follow-up), clinical location (a higher proportion were COVID-facing in the completers group vs. the non-completers group), and role (a higher proportion of registered nurses in the completers group vs. the non-completers group). All of these variables were included in the multivariable GEE model.

**Figure 1:**
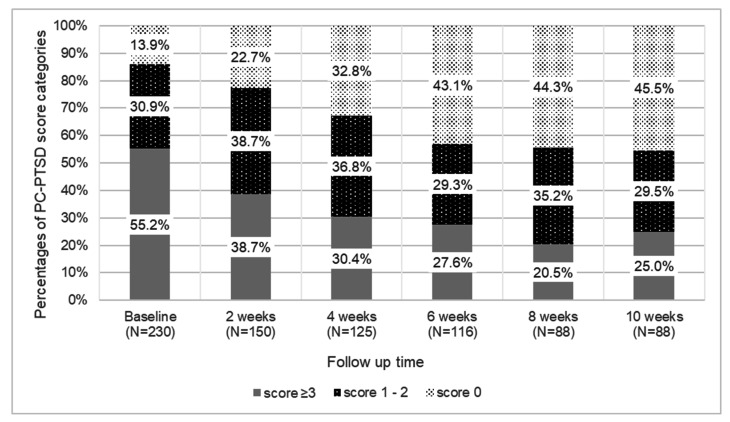
Proportion of participants with PC-PTSD scores of 0, 1–2, and ≥3 across the 10-week follow-up period.

As depicted in Figure 1, when compared to the baseline assessment, the proportion of participants enrolled in the longitudinal study with PC-PTSD scores ≥ 3 decreased across each of the two-week follow-up time periods and was lowest at week 8, before increasing again at week 10 (38.7% at week 2, 30.4% at week 4, 27.6% at week 6, 20.4% at week 8, and 25.0% at week 10). 

The change in the prevalence of positive PC-PTSD screens (PC-PTSD ≥ 3) was statistically significant between the baseline assessment and 10-week follow-up (change, 30.2%; *p* < 0.001). At the baseline assessment, 30.9% of participants had PC-PTSD scores between 1–2 and 13.9% had a PC-PTSD score of 0. At the end of the 10-week follow-up period, 45.5% of participants had a PC-PTSD score of 0 indicating no acute stress. Among those who initially had a PC-PTSD score of 0 or 1–2 at baseline (*n* = 103), the incidence of a positive screen for PTSD (score ≥ 3) was 10.5% at week 2, 11.9% at week 4, 9.5% at week 6, 8.0% at week 8, and 12.2% at week 10 (p-trend= 0.034). 

When assessing the components of the PC-PTSD questionnaire, “avoiding thinking about COVID-19” was the most prevalent symptom reported at baseline (70%), followed by “having nightmares about COVID-19” (68%), “feeling numb/detached” (60%), and being “on guard/watchful” (55%: (Supplementary Figure S1)). “Avoiding thinking about COVID-19” was also the most prevalent symptom reported at week 2 (55%), week 4 (46%), week 6 (42%), and at week 10 (38%) follow-up time periods. With the exception of week 6, “being on guard/watchful” was the least prevalent symptom reported across all time periods including at the end of 10-week follow-up period. 

In univariable analyses, being a nurse (odds ratio [OR]: 1.97, 95% confidence interval [CI]: 1.26, 3.07), female (OR: 3.60, 95% CI: 1.92, 6.72), and working in a COVID-19-focused location (OR: 1.67, 95% CI: 1.16, 2.40) were all associated with increased odds of a positive PC-PTSD screen at 10-week follow-up (Table 2). 

These same factors maintain statistical significance in the multivariable-adjusted analyses (being a nurse OR: 1.70, 95% CI: 1.06–2.71; female OR: 3.00, 95% CI: 1.59, 5.72; and working in a COVID-19-focused location OR: 1.51, 95% CI: 1.02, 2.21) (Table 2). Even though age and number of hours worked per week were not statistically significant predictors of a positive PC-PTSD screen, results showed interesting trends. Specifically, longer working hours (in 10 h increments) increased the odds of a positive PC-PTSD screen by 8% (OR: 1.08, 95% CI: 0.99–1.18), adjusting for all other covariates present in the model.

When examining a shorter follow-up period (6-week follow-up (i.e., 4 weeks after the initial baseline assessment)), results were similar to analyses for the 10-week follow-up period. Female gender (OR: 2.89, 95% CI: 1.52, 5.52) and working in a COVID-19-focused location (OR: 1.58, 95% CI: 1.03, 2.45) were associated with an increased odds of a positive PTSD screen in multivariable-adjusted analyses (Supplementary Table S3). Interestingly, at 6-week follow-up, being a nurse was associated with increased odds for a positive PTSD screen and being a white non-Hispanic/Latino healthcare worker was associated with lower odds of a positive PTSD screen only in univariable analyses. Age was not a significant predictor of the outcome in any of the models.

## 4. Discussion

In this 3-month study of NYC healthcare workers during the first wave of the COVID 19 pandemic (Spring 2020) when NYC was the center of the US pandemic, more than half of participants had elevated acute stress symptoms at baseline assessment. At the end of the 10-week follow-up period, one out of four healthcare workers had a positive screen on the PC-PTSD indicating an increased risk for PTSD. Among those without a positive screen for PTSD symptoms at baseline, 12.2% subsequently developed PTSD symptoms at 10 weeks. Almost half of participants at the end of 10-week follow-up did not report any posttraumatic stress symptoms (i.e., PC-PTSD score of 0). The most prevalent symptom was “avoiding thinking about COVID-19.” Being female was associated with three-fold higher odds of a positive screen on the PC-PTSD at 10-week follow-up. Working as a nurse and in a COVID-19-facing location also increased the odds of a positive PC-PTSD screen by 70% and 51%, respectively.

During the COVID-19 pandemic, cross-sectional studies have demonstrated that healthcare workers have high rates of positive screens for acute stress and posttraumatic symptoms. In a study of 1563 healthcare workers from Wuhan China, 73.4% reported symptoms of acute stress [1]. In a recent systematic review and meta-analysis, the pooled prevalence of acute stress was 40.3% (95% CI 31.4–50.0%, *n* = 16,235 healthcare workers) and of posttraumatic symptoms was 11.4% (95% CI 3.6–30.9, *n* = 3676) [21]. Most notably, none of these studies in that systematic review and meta-analysis included longitudinal assessments of the same cohort of healthcare workers during the COVID-19 pandemic to determine the prevalence or incidence of stress or posttraumatic symptoms at follow-up.

Because of the increased psychological distress reported among healthcare workers, there have been several calls to action to address healthcare workers’ mental health. A US Congress bill [3] advocates for federal funding to expand mental health support services for healthcare workers during the pandemic and to conduct studies to determine the short- and long-term risk of psychological distress among healthcare workers. Addressing psychological distress symptoms such as posttraumatic stress in healthcare workers is imperative, as it is associated with negative health outcomes for healthcare workers and is linked to negative patient outcomes, including increased medical errors [22,23].

Acute stress may be a precursor for the development of PTSD if symptoms persist, and may arise as early as four weeks after an acute traumatic event [24,25]. It has been hypothesized that acute stress can lead to chronic increases in plasma glucocorticoid levels and a change in the hypothalamic–pituitary–adrenal (HPA) axis and in glucocorticoid responsiveness to subsequent stressors [26]. Individuals with prior acute stress may have increased sensitivity of the HPA axis leading to enhanced sensitivity to acute stress or subsequent traumas overtime, leading to PTSD [26].

PTSD can cause physiological changes, including alterations in the immunological and autonomic nervous systems [7,27] leading to increased risk for adverse psychological outcomes, chronic diseases, and mortality [6,7,8,9,10]. For example, the Nurses’ Health Study II (*n* = 51,602) demonstrated that nurses with trauma exposure who reported 1–3 positive PTSD symptoms had a 43% increased risk for death over a 9-year follow-up period compared to nurses without trauma exposure [9]. Interestingly, data from prior infectious disease outbreaks (i.e., SARS and Middle East Respiratory Syndrome [MERS]) have provided conflicting information, with some reporting a low prevalence of PTSD symptoms, suggesting that most individuals do not develop long-term psychological distress or psychological disorders including PTSD [5], while others have reported a higher prevalence [28]. A meta-analysis of 11 studies conducted among healthcare workers during the SARS and MERS infectious disease outbreaks reported the prevalence of PTSD symptoms among healthcare workers to be 14% (95% CI 8–20, *n* = 1712) and 33% (95% CI 12–55, *n* = 682), respectively [6]. Su et al. [28] examined the prevalence of self-reported PTSD symptoms among 102 healthcare workers one month after the acute phase of the 2003 SARS outbreak in Taiwan. The prevalence of PTSD symptoms was 28.4%. Yet, few data are available on the prevalence of PTSD at follow-up in healthcare workers during the COVID-19 pandemic.

Our findings that 25% of healthcare workers had a positive PC-PTSD screen at 10-week follow-up are consistent with a study by Baumann et al. [12] of 262 Emergency Department physicians (from March–April 2020) conducted at seven institutions in California, New Jersey, and Louisiana. At baseline, 32.8% had a positive screen for acute stress (using the PC-PTSD 5 questionnaire). At follow-up (one-time repeat assessment 4–6 weeks later), 25.9% had a positive screen indicating increased risk for PTSD. However, one of the major limitations of that study is the heterogeneity in participants’ levels of COVID-19 exposure in the clinical setting and therefore in levels of acute stress, as participants were recruited from multicenter sites that were undergoing different phases of the COVID-19 pandemic. This included one study site that was still in a pre-pandemic period. Additionally, no data on incidence were provided in that study. Our study extends these findings, as it provides a longer follow-up time period, provides data on incidence of posttraumatic symptoms among healthcare workers, is inclusive of multidisciplinary healthcare workers, and enrolled participants from the same academic center who experienced the same phase of the COVID-19 pandemic. Conversely, our finding that 45.4% of participants did not have any posttraumatic stress symptoms at the 10-week follow-up period may indicate that most healthcare workers do not develop long-term psychological disorders such as PTSD, a finding that needs confirmation in future studies.

In the current analysis, being female was associated with a positive PC-PTSD screen at follow-up, a finding consistent with Baumann et al. [12] Female gender has been associated with acute stress during the COVID-19 pandemic in prior studies [12,29,30,31]. While our finding that being a nurse and working in COVID-19-facing setting are additional factors associated with increased odds of a positive PC-PTSD screen at 10-week follow-up is new, both have been reported as risk factors for acute stress in previous studies. In a study of 653 healthcare workers in Italy, nurses vs. doctors and those who worked as frontline workers were more likely to have a positive screen for PTSD [31]. Nurses may be particularly at risk for PTSD during the COVID-19 pandemic, as they spend more time delivering direct patient care. Additionally, Lai et al. demonstrated that among 1257 healthcare workers in China, frontline work was associated with 1.6-fold higher odds of acute distress [29]. Our finding that working in a COVID-19-facing location is a risk factor for a positive PC-PTSD screen is not surprising, as this may be one of the “acute traumatic events” for healthcare workers. Nonetheless, these findings will need to be confirmed in future studies. Lastly, while there are currently no guidelines established for interventions to decrease the incidence of acute stress and/or PTSD among healthcare workers during the COVID-19 pandemic, it is imperative to consider practical implications of this study including possible interventions. Several studies [32,33] are investigating treatment regimens and protocols. One such example is the REduction of STress (REST) randomized controlled trial [33] which is evaluating the effect of an online cognitive behavior treatment program vs. control conditions on stress and incident psychiatric disorders at 3- and 6-month follow-up for healthcare workers during the COVID-19 pandemic.

### Strengths and Limitations

The current study has several strengths. It included a large sample of healthcare workers with different medical backgrounds, including nurses, physicians, and advanced practice providers. Our study provides one of the few published reports examining the prevalence and incidence of a positive PC-PTSD screen in a longitudinal follow-up study among healthcare workers working in a US center during the first wave of the COVID-19 pandemic. However, there are several limitations to this study, including small sample size and attrition. Only 88 participants completed all assessments at 10 weeks. Therefore, subgroup analyses could not be performed. Our study may also be limited by selection bias. Compared to our baseline study, only 27.8% decided to enroll in the longitudinal study. Nonetheless, there were no significant differences in demographic characteristics or baseline acute stress symptoms among those who participated in the longitudinal study and those who did not. Our participants were recruited from one large academic center, and therefore may not be representative of participants from other healthcare systems. Lastly, our assessments of acute stress and PTSD symptoms were based only on self-report, could be impacted by information bias, and were not clinically confirmed by a medical professional for the diagnosis of PTSD.

## 5. Conclusions

In summary, acute stress was prevalent at baseline assessment among NYC healthcare workers enrolled in a 10-week study during the first wave of the COVID-19 pandemic. One out of four healthcare workers in our study screened positive for posttraumatic stress symptoms at 10-week follow-up, indicating an increased risk for PTSD. Twelve percent of healthcare workers who at baseline had a negative screen on the PC-PTSD questionnaire subsequently developed PTSD symptoms at 10 weeks. Encouragingly, almost half of participants did not have any posttraumatic symptoms at the end of the follow-up period. Being female, a nurse, and working in a COVID-19-facing location were associated with higher odds of a positive PC-PTSD screen at 10-week follow-up. Our study provides new insights into the long-term effects of the COVID-19 pandemic on healthcare workers’ mental health. Further research is needed on the impact of the continued pandemic, which has now lasted for more than one year, as well as subsequent waves of the pandemic, on acute stress and development of PTSD among healthcare workers.

## Figures and Tables

**Table 1:** Baseline characteristics of participants who agreed to participate in the longitudinal follow-up assessments (N = 230).

	(N = 230)
Age (years), median (IQR)	36 (31–48)
Sex (N %)	
Female	183 (79.6%)
Male	46 (20.0%)
Other	1 (0.4%)
Race (N %)	
White	148 (64.3%)
Asian	26 (11.3%)
Black	21 (9.1%)
Other	20 (8.7%)
More than one race	14 (6.1%)
Hawaiian/Pacific Islander	1 (0.4%)
American Indian/Native American	0 (0%)
Ethnicity (N %)	
Not Hispanic or Latino	191 (86.8%)
Hispanic or Latino	29 (13.2%)
Prefer not to answer	10 (4.3%)
Clinical location (N %)	
COVID-facing	190 (82.6%)
Not COVID-facing	40 (17.4%)
Hours worked in past week (at baseline) ^a^	
Median (IQR)	41–50 h (31–40 h, 51–60 h)
Role (N %)	
Registered Nurse	115 (50.0%)
Attending Physician	50 (21.7%)
Resident/Fellow	43 (18.7%)
Advanced Practice Provider	13 (5.7%)
Other	8 (3.5%)
Prefer not to answer	1 (0.4%)

^a^ Selections were based on 13 categories: 0–10 h, 11–20 h, 21–30 h, 31–40 h, 41–50 h, 51–60 h, 61–70 h, 71–80 h, 91–100 h, 101–110 h, 111–120 h, 120+ h.

**Table 2:** Generalized estimating equation (GEE) univariable and multivariable models to identify factors associated with PC-PTSD score ≥ 3 at 10-week follow-up.

	Univariable Model			Multivariable Model		
Variable	B (SE)	OR (95% CI)	*p*-Value	B (SE)	Adjusted OR (95% CI)	*p*-Value
Age	−0.02 (0.01)	0.98 (0.96, 1.00)	0.061	−0.02 (0.01)	0.98 (0.96, 1.00)	0.089
Role (RN vs. other)	0.68 (0.23)	1.97 (1.26, 3.07)	0.003	0.53 (0.24)	1.70 (1.06, 2.71)	0.028
Sex (female vs. male)	1.28 (0.32)	3.60 (1.92, 6.72)	<0.001	1.10 (0.33)	3.00 (1.59, 5.72)	0.001
Clinical location(COVID-facing vs. not)	0.51 (0.18)	1.67 (1.16, 2.40)	0.005	0.41 (0.20)	1.51 (1.02, 2.21)	0.037
Work hours ^a^	0.05 (0.04)	1.05 (0.97, 1.13)	0.217	0.08 (0.04)	1.08 (0.99, 1.18)	0.070
Race/ethnicity (White, Non-Hispanic/Latino vs. other)	−0.41 (0.23)	0.66 (0.42, 1.05)	0.077	−0.27 (0.24)	0.76 (0.47, 1.22)	0.255

B (SE): regression coefficient (standard error), OR (95% CI): odds ratio and 95% confidence interval; PC-PTSD: primary care posttraumatic stress disorder; RN: registered nurse. ^a^ Coefficients and ORs for “Work Hours” were calculated for 10 h increments across the categories: 0–10 h, 11–20 h, 21–30 h, 31–40 h, 41–50 h, 51–60 h, 61–70 h, 71–80 h, 91–100 h, 101–110 h, 111–120 h, 120+ h.

## Data Availability

The data presented in this study are available on request to the corresponding author.

## Supplementary Materials

https://www.mdpi.com/article/10.3390/ijerph19010262/s1, Figure S1: PC-PTSD Questionnaire Symptom Domains across 10-week Follow-Up Period; Table S1: Baseline characteristics of participants who agreed to participate in the longitudinal follow-up assessments (N = 230) and those who did not agree to participate (N = 597); Table S2: Baseline characteristics of participants who completed the 10-week follow-up assessment (N = 88) and those who did not complete the 10-week follow-up assessment (N = 142); Table S3: Generalized estimating equation (GEE) univariable and multivariable models to identify factors associated with PC-PTSD score ≥ 3 at 6-week follow-up.

## References

[1] Zhang C., Yang L., Liu S., Ma S., Wang Y., Cai Z., Du H., Li R., Kang L., Su M. (2020). Survey of Insomnia and Related Social Psychological Factors Among Medical Staff Involved in the 2019 Novel Coronavirus Disease Outbreak. Front. Psychiatry.

[2] WHO Keep Health Workers Safe to Keep Patients Safe: WHO. https://www.who.int/news/item/17-09-2020-keep-health-workers-safe-to-keep-patients-safe-who.

[3] S.610—Dr Lorna Breen Health Care Provider Protection Act. https://www.congress.gov/bill/117th-congress/senate-bill/610?q=%7B%22search%22%3A%5B%22S.+610%22%5D%7D&s=7&r=1.

[4] Pfefferbaum B., North C.S. (2020). Mental Health and the Covid-19 Pandemic. N. Engl. J. Med..

[5] Maunder R.G., Lancee W.J., Balderson K.E., Bennett J.P., Borgundvaag B., Evans S., Fernandes C.M.B., Goldbloom D.S., Gupta M., Hunter J.J. (2006). Long-term Psychological and Occupational Effects of Providing Hospital Healthcare during SARS Outbreak. Emerg. Infect. Dis..

[6] Salehi M., Amanat M., Mohammadi M., Salmanian M., Rezaei N., Saghazadeh A., Garakani A. (2021). The prevalence of post-traumatic stress disorder related symptoms in Coronavirus outbreaks: A systematic-review and meta-analysis. J. Affect. Disord..

[7] Aresnson B., Cohen B. Posttraumatic Stress Disorder and Cardiovascular Disease. https://www.ptsd.va.gov/publications/rq_docs/V28N1.pdf.

[8] Giesinger I., Li J., Takemoto E., Cone J.E., Farfel M.R., Brackbill R.M. (2020). Association Between Posttraumatic Stress Disorder and Mortality Among Responders and Civilians Following the September 11, 2001, Disaster. JAMA Netw. Open.

[9] Roberts A.L., Kubzansky L.D., Chibnik L.B., Rimm E.B., Koenen K.C. (2020). Association of Posttraumatic Stress and Depressive Symptoms With Mortality in Women. JAMA Netw. Open.

[10] Sumner J.A., Kubzansky L.D., Elkind M.S., Roberts A.L., Agnew-Blais J., Chen Q., Cerda M., Rexrode K.M., Rich-Edwards J.W., Spiegelman D. (2015). Trauma Exposure and Posttraumatic Stress Disorder Symptoms Predict Onset of Cardiovascular Events in Women. Circulation.

[11] Breslau N., Peterson E.L., Schultz L.R. (2008). A Second Look at Prior Trauma and the Posttraumatic Stress Disorder Effects of Subsequent Trauma: A Prospective Epidemiological Study. Arch. Gen. Psychiatry.

[12] Baumann B.M., Cooper R.J., Medak A.J., Lim S., Chinnock B., Frazier R., Roberts B.W., Epel E.S., Rodriguez R.M. (2021). Emergency physician stressors, concerns, and behavioral changes during COVID-19: A longitudinal study. Acad. Emerg. Med..

[13] Magnavita N., Soave P.M., Antonelli M. (2021). A One-Year Prospective Study of Work-Related Mental Health in the Intensivists of a COVID-19 Hub Hospital. Int. J. Environ. Res. Public Health.

[14] New York City Region Is Now an Epicenter of the Coronavirus Pandemic. https://www.nytimes.com/2020/03/22/nyregion/Coronavirus-new-York-epicenter.html?.

[15] Shechter A., Diaz F., Moise N., Anstey D.E., Ye S., Agarwal S., Birk J.L., Brodie D., Cannone D.E., Chang B. (2020). Psychological distress, coping behaviors, and preferences for support among New York healthcare workers during the COVID-19 pandemic. Gen. Hosp. Psychiatry.

[16] COVID-19 Peak Dates: Updated Projections for Each State. https://www.beckershospitalreview.com/patient-flow/covid-19-peak-dates-updated-projections-for-each-state.html.

[17] What Does a Coronavirus Peak in New York Mean?. https://www.wsj.com/articles/what-does-a-coronavirus-peak-in-new-york-mean-11586338201.

[18] Lockdown Extended for Most of Coronavirus-Battered New York. https://www.cbsnews.com/news/new-york-stay-at-home-extended-coronavirus-lockdown/.

[19] Kumaraiah D., Yip N., Ivascu N., Hill L. Innovative ICU Physician Care Models: Covid-19 Pandemic at NewYork-Presbyterian. https://catalyst.nejm.org/doi/full/10.1056/CAT.20.0158?tk=eo_7f79f000-09b6-4575-9694-f4aeb2a2d038_fZ7gXJKpNJb2QTz1Mj6mycNPIXyRLUegXqx8.

[20] Prins A., Ouimette P., Kimerling R., Camerond R.P., Hugelshofer D.S., Shaw-Hegwer J., Thrailkill A., Gusman F.D., Sheikh J. (2004). The primary care PTSD screen (PC–PTSD): Development and operating characteristics. Prim. Care Psychiatry.

[21] Batra K., Singh T.P., Sharma M., Batra R., Schvaneveldt N. (2020). Investigating the Psychological Impact of COVID-19 among Healthcare Workers: A Meta-Analysis. Int. J. Environ. Res. Public Health.

[22] Melnyk B.M., Orsolini L., Tan A., Arslanian-Engoren C., Melkus G.D., Dunbar-Jacob J., Rice V.H., Millan A., Dunbar S.B., Braun L.T. (2018). A National Study Links Nurses’ Physical and Mental Health to Medical Errors and Perceived Worksite Wellness. J. Occup. Environ. Med..

[23] Trockel M.T., Menon N.K., Rowe S.G., Stewart M.T., Smith R., Lu M., Kim P.K., Quinn M.A., Lawrence E., Marchalik D. (2020). Assessment of Physician Sleep and Wellness, Burnout, and Clinically Significant Medical Errors. JAMA Netw. Open.

[24] Grinage B.D. (2003). Diagnosis and management of post-traumatic stress disorder. Am. Fam. Physician.

[25] Treatment of Acute Stress Disorder in Adults. https://www.uptodate.com/contents/treatment-of-acute-stress-disorder-in-adults.

[26] Elzinga B.M., Schmahl C., Vermetten E., Van Dyck R., Bremner J.D. (2003). Higher Cortisol Levels Following Exposure to Traumatic Reminders in Abuse-Related PTSD. Neuropsychopharmacology.

[27] Brudey C., Park J., Wiaderkiewicz J., Kobayashi I., Mellman T.A., Marvar P.J. (2015). Autonomic and inflammatory consequences of posttraumatic stress disorder and the link to cardiovascular disease. Am. J. Physiol. Integr. Comp. Physiol..

[28] Su T.-P., Lien T.-C., Yang C.-Y., Su Y.L., Wang J.-H., Tsai S.-L., Yin J.-C. (2007). Prevalence of psychiatric morbidity and psychological adaptation of the nurses in a structured SARS caring unit during outbreak: A prospective and periodic assessment study in Taiwan. J. Psychiatr. Res..

[29] Lai J., Ma S., Wang Y., Cai Z., Hu J., Wei N., Wu J., Du H., Chen T., Li R. (2020). Factors associated with mental health outcomes among health care workers exposed to coronavirus disease 2019. JAMA Netw. Open.

[30] Elbay R.Y., Kurtulmuş A., Arpacıoğlu S., Karadere E. (2020). Depression, anxiety, stress levels of physicians and associated factors in Covid-19 pandemics. Psychiatry Res..

[31] Bassi M., Negri L., Delle Fave A., Accardi R. (2020). The relationship between post-traumatic stress and positive mental health symptoms among health workers during COVID-19 pandemic in Lombardy, Italy. J. Affect. Disord..

[32] Bureau R., Bemmouna D., Faria C.G.F., Goethals A.-A.C., Douhet F., Mengin A.C., Fritsch A., Bertschy A.Z., Frey I., Weiner L. (2021). My Health Too: Investigating the Feasibility and the Acceptability of an Internet-Based Cognitive-Behavioral Therapy Program Developed for Healthcare Workers. Front. Psychol..

[33] Weiner L., Berna F., Nourry N., Severac F., Vidailhet P., Mengin A.C. (2020). Efficacy of an online cognitive behavioral therapy program developed for healthcare workers during the COVID-19 pandemic: The REduction of STress (REST) study protocol for a randomized controlled trial. Trials.

