# Supplementary material for: Prevalence, Incidence, and Factors Associated with Posttraumatic Stress at Three-Month Follow-Up among New York City Healthcare Workers after the First Wave of the COVID-19 Pandemic
Source: Int J Environ Res Public Health. 2021 Dec 27;19(1):262. doi: 10.3390/ijerph19010262 (PMC8750525; doi:10.3390/ijerph19010262)
Supplement: Supplementary file 1 [file ijerph-19-00262-s001.zip › ijerph-1489674-supplementary.pdf]

Supplement

Supplementary Figure S1: PC-PTSD Questionnaire Symptom Domains across 10-week Follow-Up Period

Supplementary Table S1: Baseline characteristics of participants who agreed to participate in the longitudinal follow-up assessments (N=230) and those who did not agree to participate (N=597)

Supplementary Table S2: Baseline characteristics of participants who completed the 10-week follow-up assessment (N=88) and those who did not complete the 10-week follow-up assessment (N=142)

Supplementary Table S3: Generalized estimating equation (GEE) univariable and multivariable models to identify factors associated with PC-PTSD score ≥ 3 at 6-week follow-up

Supplementary Figure S1:

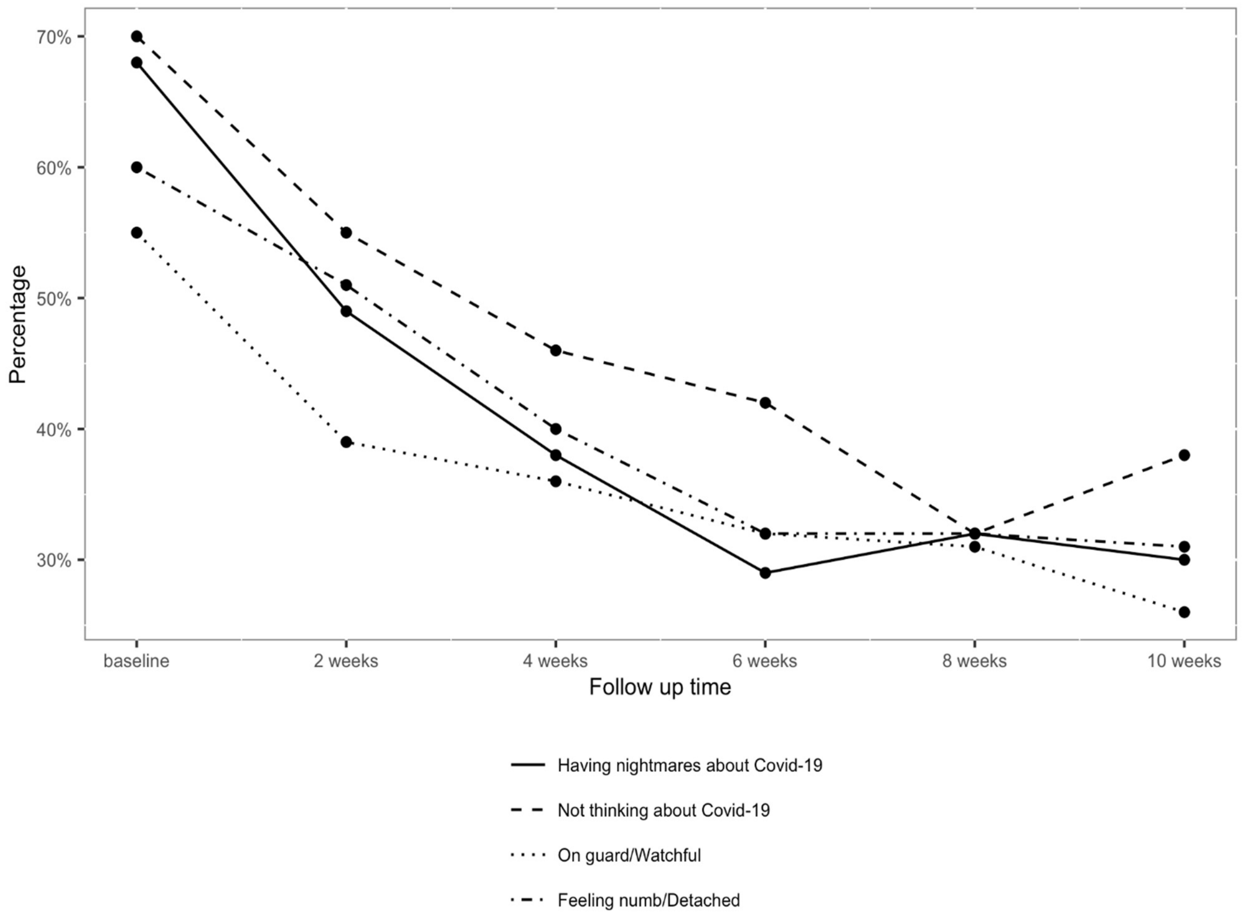

Supplementary Table S1: Baseline characteristics of participants who agreed to participate in the longitudinal follow-up assessments (N=230) and those who did not agree to participate (N=597)

	Agreed to participate (N=230)	Did not agree to participate (N=597)	P-value[c]
**Sex** (N %)			0.271
Female	183 (79.6%)	474 (80.6%)	
Male	46 (20.0%)	114 (19.4%)	
Other	1 (0.4%)	0 (0%)	
**Race** (N %)			0.159
White	148 (64.3%)	334 (55.9%)	
Asian	26 (11.3%)	105 (17.6%)	
Black	21 (11.3%)	47 (7.9%)	
Other	20 (8.7%)	68 (11.4%)	
More than one race	14 (6.1%)	36 (6.0%)	
Hawaiian/Pacific Islander	1 (0.4%)	3 (0.5%)	
American Indian/Native American	0 (%)	4 (0.7%)	
**Ethnicity** (N %)			0.784
Not Hispanic or Latino	191 (86.8%)	485 (87.5%)	
Hispanic or Latino	29 (13.2%)	69 (12.5%)	
**Clinical location** (N %)			0.468
COVID-19 facing	190 (82.6%)	480 (80.4%)	
Not COVID-19 facing	40 (17.4%)	117 (19.6%)	
**Hours worked in past week (at baseline)[a]**			0.093
Median (IQR)	41-50 hrs (31-40 h, 51-60 h)	41-50 hrs (31-40 h, 51-60 h)	
**Role** (N %)			0.077
Registered Nurse	115 (50.0%)	347 (58.1%)	
Attending Physician	50 (21.7%)	95 (15.9%)	
Resident/Fellow	43 (18.7%)	98 (17.9%)	
Advanced Practice Provider	13 (5.7%)	48 (8.0%)	
Other	8 (3.5%)	6 (1.0%)	
Prefer not to answer	1 (0.4%)	3 (0.5%)	
**Acute stress/PC-PTSD score[b]**			0.116
Median (IQR)	3.00 (1.00, 4.00)	3.00 (2.00, 4.00)	
**Dichotomized acute stress/PC-PTSD score**			0.307
Moderate or severe acute stress/PTSD (positive screen, ≥ 3 score)	127 (55.2%)	353 (59.1%)	
None or mild acute stress/PTSD (< 3 score)	103 (44.8%)	244 (40.9%)	

[a] Selections were based on 13 categories: 0-10h, 11-20h, 21-30h, 31-40h, 41-50h, 51-60h, 61-70h, 71-80h, 91-100h, 101-110h, 111-120h, 120+h

[b] Ratings were based on the 4-item Primary Care PTSD screen (range 0-4)

[c] P-values are based on chi-squared/Fisher Exact test for categorical variables and Wilcoxon Rank-Sum test for continuous variables

PC-PTSD: primary care posttraumatic stress disorder

Supplementary Table S2: Baseline characteristics of participants who completed the 10-week follow-up assessment (N=88) and those who did not complete the 10-week follow-up assessment (N=142)

	**Did not complete 10-week assessment (N=142)**	**Completed 10-week assessment (N=88)**	**P-value[b]**
**Age (yrs),** median (IQR)	35 (31 – 44.3)	41 (32 - 53)	0.004
**Sex** (N %)			0.408
Female	114 (80.9%)	69 (77.5%)	
Male	27 (19.1%)	19 (21.3%)	
Other	0 (0%)	1 (1.1%)	
**Race** (N %)			0.334
White	84 (59.6%)	64 (71.9%)	
Asian	16 (11.3%)	10 (11.2%)	
Black	14 (9.9%)	7 (7.9%)	
Other	16 (8.7%)	4 (4.5%)	
More than one race	10 (7.1%)	4 (4.5%)	
Hawaiian/Pacific Islander	1 (0.7%)	0 (0%)	
American Indian/Native American	0 (%)	0 (0%)	
**Ethnicity** (N %)			0.302
Not Hispanic or Latino	118 (83.7%)	73 (82%)	
Hispanic or Latino	23 (16.3%)	16 (18%)	
**Clinical location (N %)**			0.020
COVID-facing	123 (87.2%)	67 (75.3%)	
Not COVID-facing	18 (12.8%)	22 (24.7%)	
**Hours worked in past week (at baseline)[a]**			0.348
Median (IQR)	41-50 hrs (31-40 h, 51-60 h)	41-50 hrs (31-40 h, 51-60 h)	
**Role** (N %)			0.009
Registered Nurse	78 (55.3%)	37 (41.6%)	
Attending Physician	19 (13.5%)	30 (33.7%)	
Resident	23 (16.3%)	8 (9%)	
Advanced Practice Provider	0 (0%)	1 (1.1%)	
Fellow	9 (6.4%)	3 (3.4%)	
Other	11 (7.8%)	10 (11.2%)	
Prefer not to answer	1 (0.7%)	0 (0%)	

[a] Selections were based on 13 categories: 0-10h, 11-20h, 21-30h, 31-40h, 41-50h, 51-60h, 61-70h, 71-80h, 91-100h, 101-110h, 111-120h, 120+h

[b] P-values are based on chi-squared/Fisher Exact test for categorical variables and Wilcoxon Rank-Sum test for continuous variables

Supplementary Table S3: Generalized estimating equation (GEE) univariable and multivariable models to identify factors associated with PC-PTSD score ≥ 3 at 6-week follow-up

	Univariable model			Multivariable model		
**Variable**	**B (SE)**	**OR (95% CI)**	**P-value**	**B (SE)**	**Adjusted OR (95% CI)**	**P-value**
**Age**	-0.02 (0.01)	0.98 (0.96, 1.00)	0.061	-0.02 (0.01)	0.98 (0.96, 1.00)	0.083
**Role** (RN vs. other)	0.60 (0.24)	1.82 (1.13, 2.92)	**0.013**	0.43 (0.26)	1.54 (0.92, 2.55)	0.097
**Sex** (female vs. male)	1.22 (0.32)	3.39 (1.80, 6.37)	**<0.001**	1.06 (0.33)	2.89 (1.52, 5.52)	**0.001**
**Clinical location** (COVID-facing vs. not)	0.57 (0.21)	1.77 (1.17, 2.69)	**0.007**	0.46 (0.22)	1.58 (1.03, 2.45)	**0.035**
**Work hours**[a]	0.03 (0.04)	1.03 (0.96, 1.11)	0.427	0.06 (0.04)	1.06 (0.98, 1.15)	0.128
**Race/ethnicity** (White, non-Hispanic/Latino vs. other)	-0.48 (0.24)	0.62 (0.39, 0.98)	**0.042**	-0.36 (0.25)	0.70 (0.43, 1.13)	0.146

B (SE): regression coefficient (standard error), OR (95% CI): odds ratio and 95% confidence interval; PC-PTSD: primary care posttraumatic stress disorder; RN: registered nurse

[a]Coefficients and ORs for “Work Hours” were calculated for 10h increments across the categories: 0-10h, 11-20h, 21-30h, 31-40h, 41-50h, 51-60h, 61-70h, 71-80h, 91-100h, 101-110h, 111-120h, 120+h
